# Genetic Analyses of Cell-Free DNA in Pancreatic Juice or Bile for Diagnosing Pancreatic Duct and Biliary Tract Strictures

**DOI:** 10.3390/diagnostics12112704

**Published:** 2022-11-05

**Authors:** Kosuke Nagai, Masaki Kuwatani, Koji Hirata, Goki Suda, Hajime Hirata, Yunosuke Takishin, Ryutaro Furukawa, Kazuma Kishi, Hiroki Yonemura, Shunichiro Nozawa, Ryo Sugiura, Kazumichi Kawakubo, Naoya Sakamoto

**Affiliations:** 1 Department of Gastroenterology and Hepatology, Hokkaido University Hospital, Sapporo 060-8648, Japan; 2 Department of Gastroenterology and Hepatology, Hakodate Municipal Hospital, Hakodate 041-8680, Japan

**Keywords:** bile, bile duct, cell-free DNA, liquid biopsy, mutation, pancreatic duct, pancreatic juice, stricture

## Abstract

Poor prognosis of pancreaticobiliary malignancies is attributed to intrinsic biological aggressiveness and the lack of reliable methods for early diagnosis. This study aimed to evaluate the feasibility and availability of pancreatic juice- and bile-derived cell-free DNA (cfDNA) for diagnosing pancreaticobiliary strictures. From October 2020 to February 2022, pancreatic juice or bile was obtained from 50 patients with pancreaticobiliary strictures during endoscopic retrograde cholangiopancreatography. cfDNAs extracted from the samples were analyzed using next-generation sequencing and a cancer gene panel. The obtained cfDNAs, genetic data and clinical information were analyzed for diagnosis. cfDNA concentrations in pancreatic juice were higher in the intraductal papillary mucinous neoplasm group than in the other groups, whereas those in bile were similar in all groups. In pancreatic juice, the sensitivity, specificity and positive and negative predictive values of cfDNA analyses were 33%, 100%, 100% and 71.4%, respectively, whereas those of cytological analyses were 0%, 100%, 0% and 62.5%, respectively. In bile, those of cell-free DNA analyses were 53%, 75%, 89.5% and 28.6%, respectively, whereas those of cytological analyses were 19%, 100%, 100% and 16%, respectively. In conclusion, pancreatic juice- and bile-derived cfDNA is a novel liquid biopsy tool that can diagnose pancreaticobiliary strictures.

## 1. Introduction

Pancreatic ductal adenocarcinoma (PDAC) and biliary tract cancer (BTC), including ampullary cancer, cholangiocarcinoma, and gallbladder carcinoma, are aggressive malignant tumors, and their five-year survival rates are extremely low compared to those of other malignancies [1,2]. Although surgical resection is the only reliable treatment for a complete cure, many patients with pancreaticobiliary malignancies are diagnosed with unresectable conditions [3,4]. Their poor prognoses are attributed to intrinsic biological aggressiveness, late clinical symptoms and a lack of reliable methods for early diagnosis [5,6]. Although PDAC and BTC are often associated with pancreatic duct and biliary tract strictures, some benign diseases, such as IgG4-related sclerosing cholangitis/pancreatitis, chronic pancreatitis and biliary stone-related cholangitis, can also cause these strictures, making the diagnosis difficult. 

Endoscopic ultrasound-guided fine needle aspiration (EUS-FNA) has been performed for pancreaticobiliary diseases with a mass formation in the pancreas or biliary tract, and its good diagnostic performance has been reported [7]. Additionally, endoscopic retrograde cholangiopancreatography (ERCP) with forceps biopsy and bile/pancreatic juice cytology or brushing cytology has been conventionally performed for diagnosing pancreaticobiliary diseases showing pancreatic duct or bile duct strictures. However, ERCP sensitivity to malignancy is insufficient and low, ranging from 30% to 70% [8,9,10,11,12,13]. Thus, patients may require repeated ERCP, leading to delayed diagnosis and treatment or unnecessary surgery. 

Liquid biopsy has recently sparked attention as a promising tool for cancer detection and is a new diagnostic technique that measures the cell-free DNA (cfDNA) derived from cancer cells in blood and body fluids [14,15]. However, the diagnostic value of blood cfDNA for PDAC and BTC remains limited [16,17]. Theoretically, pancreatic juice or bile directly contacts the tumor cells in PDAC and BTC and should harbor abundant tumor-derived materials, such as cfDNA, exosomes and metabolomes. Therefore, more cfDNA would be suspended in pancreatic juice and bile than in blood; thus, pancreatic juice or bile would be a useful and available material for liquid biopsy. Previous studies have analyzed the genetic mutational profiles of PDAC and BTC using surgical specimens and found that *KRAS*, *TP53*, *SMAD4* and *CDKN2A* in PDAC and *TP53*, *KRAS*, *SMAD4*, *EGFR* and *ARID1* in BTC are important mutational genes [18,19]. Few studies have assessed the diagnostic and prognostic values of cfDNA in pancreatic juice and bile for PADC and BTC, and some earlier studies have supported the possibility of improving the diagnosis of malignancies [20,21,22,23,24,25,26]. Mateos et al. [20] reported the presence of *TP53*/*KRAS* mutations in pancreatic juice-derived cfDNA for detecting PDAC concomitant with intraductal papillary mucinous neoplasm (IPMN)/malignant IPMN. Arechederra et al. [26] reported that sensitivity to malignancy of next-generation sequencing (NGS) assay with bile cfDNA was 100% in patients with an initial diagnosis of benign (*KRAS*, 8/11; *TP53*, 5/11; *ERBB3*, 4/11 cases) or indeterminate strictures (*KRAS*, 4/7; *TP53*, 4/7 cases), in addition to a total sensitivity of 96.4% using a 161-gene panel. Moreover, ERCP is performed to relieve pancreatic and biliary obstructions, in addition to work-up, and pancreatic juice and bile are easily obtained during the procedure.

Thus, this study aimed to evaluate the feasibility and availability of cfDNA derived from pancreatic juice and bile obtained from patients with pancreatic/bile duct strictures using NGS technology with a cancer-related gene panel as a useful and alternative diagnostic tool for pancreaticobiliary diseases.

## 2. Materials and Methods

### 2.1. Patients and Samples

From October 2020 to February 2022, pancreatic juice or bile was prospectively obtained from 50 patients with pancreatic duct or bile duct stricture at Hokkaido University Hospital. The inclusion criteria were as follows: (1) having pancreatic duct or bile duct stricture based on ultrasonography, computed tomography (CT), magnetic resonance imaging/magnetic resonance cholangiopancreatography or endoscopic ultrasonography, which requires ERCP-related procedures; (2) aged 20 years or older; and (3) willingness to provide an informed consent. The exclusion criteria were as follows: (1) inability to obtain an adequate sample and (2) unsuitable for inclusion at the discretion of the principal investigator. All patients required ERCP for the diagnosis and treatment of pancreatic/bile duct stricture. Pancreatic juice was obtained from 10 patients (pancreatic juice group) and bile from 40 patients (bile group). In addition, paired peripheral blood samples from the same patients were collected. Peripheral blood mononuclear cells were isolated by centrifugation and served as the control in the NGS assay. All patients provided a written informed consent for the examination and use of their samples and clinical data, respectively. Final diagnoses were based on the pathological assessment of EUS-FNA, forceps biopsy and surgical specimens by expert pathologists, or by follow-up observation for more than six months. Malignancies were defined as pathological or apparent progressive diseases, such as size increase or metastasis on imaging examinations.

### 2.2. ERCP Procedures and Sample Preservation

ERCP was performed on patients with pancreatic/bile duct stricture using a duodenoscope (TJF 260V; Olympus Medical Systems, Tokyo, Japan). After wire-guided cannulation using a dedicated catheter (ERCP-catheter; MTW Endoskopie Manufaktur, Wesel, Germany) loaded with a 0.025-inch guidewire, a few milliliters of pancreatic juice or bile were aspirated, collected and immediately preserved in a cfDNA collection tube (Roche Diagnostics K.K., Basel, Switzerland) and a sterilized conical tube (MIC SCIENCE Ltd., Sapporo, Japan) for analysis and cytology, respectively, before pancreatography or cholangiography by contrast medium injection. Those tubes were immediately transferred to a 4 °C refrigerator for transient storage and thereafter appropriately processed within 1–2 h. When an endoscopic nasal pancreatic/biliary drainage tube (ENPD/ENBD) was placed, the pancreatic juice/bile were aspirated and collected via an ENPD/ENBD tube. Then, after the clinically necessary diagnostic samples were taken, research samples were obtained.

First, the samples for cfDNA analysis were centrifuged at 1600× *g* for 15 min at 22–24 °C. The supernatants were then carefully transferred to 2-mL tubes (Eppendorf) within 1 h after sample collection. Then, the samples were centrifuged at 16,000× *g* for 10 min at 22–24 °C, and the supernatants were carefully collected. After centrifugation, all samples were stored at −80°C until use. The cfDNA was extracted from pancreatic juice and bile using a Cobas^®^ cfDNA Sample Preparation Kit (Roche Diagnostics K.K.). Concentrations of cfDNA were measured using a Qubit^®^ dsDNA HS Assay kit (Life Technologies; Thermo Fisher Scientific, Inc., Waltham, MA, USA), and cfDNA fragment size distributions were analyzed using an Agilent 2100 Bioanalyzer (Agilent Technologies, Inc., Santa Clara, CA, USA). Genomic DNA (gDNA) was extracted from the blood samples using an All-Prep DNA/RNA/Protein Mini Kit (Qiagen, Valencia, CA, USA) and purified using AMPure XP (Beckman Coulter, Brea, CA, USA) and 70% ethanol; its concentrations were measured in addition to cfDNA.

### 2.3. Library Preparation

Ten nanograms of cfDNA and gDNA were used for library construction using the Ion Ampliseq Cancer Hotspot Panel v2 (Life Technologies), which targets 2790 catalogue of somatic mutations in cancer alteration hotspots in 50 cancer-related genes: *KRAS*, *NRAS*, *TP53*, *BRAF*, *FGFR1*, *FGFR2*, *FGFR3*, *IDH1*, *IDH2*, *SMAD4*, *EGFR*, *PIK3CA*, *CDKN2A*, *HRAS*, *ATM*, *RET*, *PTEN*, *PTPN11*, *HNF1A*, *FLT3*, *RB1*, *AKT1*, *CDH1*, *ERBB2*, *ERBB4*, *STK11*, *JAK2*, *JAK3*, *ALK*, *SRC*, *GNAS*, *SMARCB1*, *VHL*, *MLH1*, *CTNNB1*, *PDGFRA*, *KIT*, *KDR*, *FBXW7*, *APC*, *CSF1R*, *NPM1*, *MPL*, *MET*, *SMO*, *ABL1*, *NOTCH1*, *EZH2*, *GNA11* and *GNAQ*.

### 2.4. Emulsion Polymerase Chain Reaction and Ion Torrent Personal Genome Machine Sequencing

Pooled 100 pmol/L libraries were clonally amplified using the Ion OneTouch 2 (OT2) instrument with the Ion Personal Genome Machine Template OT2 200kit (Life Technologies, Carlsbad, CA, USA). The samples were then enriched using Ion OneTouch ES (Life Technologies). The enriched templates were loaded onto an Ion 318 chip and sequenced using the Ion Personal Genome Machine System (Life Technologies), according to the manufacturer’s protocol. The human genome build 19 was used as a reference, and variant calls and annotations were generated using the Ion Torrent Suite v5.6 software (Life Technologies). When matched normal controls were unavailable, other control sequence data (Thermo Fisher Scientific) were used. Somatic alterations were detected using statistical approaches in target and blood samples from the AmpliSeq target-blood pair workflow with customized filters. The analysis of normal paired samples is useful to minimize sequencing noise and to identify pathogenic alterations more accurately. Furthermore, the analysis helps to identify whether there are somatic or germline alterations in the genes [27]. Filters included removal of common single nucleotide polymorphisms, no impact events (synonymous, intron or reference allele) and mutation allele frequencies lower than 2% (recommended by the manufacturer protocol). The filter threshold covered 99% of the captured region in each case.

### 2.5. Definition of Malignancy

Final diagnoses were performed based on the pathological assessment of EUS-FNA, forceps biopsy and surgical specimens by expert pathologists or image findings on CT scan/ultrasonography/magnetic resonance imaging with follow-up observation for more than six months. A malignant disease was defined as pathological malignancy or apparent progressive disease, such as size increase or metastasis on imaging examinations.

In PDAC, mutations in *KRAS*, *p53*, *CDKN2A* and *SMAD4* occur at high rates [18]. Therefore, malignancy was defined as having one or more gene mutations in the four genes detected in pancreatic juice/bile. In BTC, many driver genes, such as *EGFR2*, *IDH1/2*, *PRKACA/PRKACB*, *EGFR* and *PTEN*, in each biliary site have been reported [19]. Thus, malignancy was defined as having one or more gene mutations in the 50-gene panel, as described above. If the gene mutation was not detected in pancreatic juice/bile, the pancreaticobiliary stricture was defined as a benign disease.

Cytological evaluation of malignancy in pancreatic juice and bile was performed by expert pathologists who were blinded to the final diagnoses.

### 2.6. Statistical Analysis

The Kruskal–Wallis test was used to compare quantitative data. Mcnemar’s test was performed to compare sensitivities and specificities for the diagnosis of malignancy. *p*-values < 0.05 were considered significant. All statistical analyses were performed using the free software EZR version 1.40 accessed on 1 November 2019 (https://www.jichi.ac.jp/saitama-sct/SaitamaHP.files/statmedEN.html) [28].

## 3. Results

### 3.1. Patient Characteristics in the Pancreatic Juice and Bile Groups

In the pancreatic juice group, the median age of the patients was 65.5 years (range, 47–80 years), and seven (70%) were men. Pancreatic diseases were described as PDAC (1/10, 10%); high-grade pancreatic intraepithelial neoplasm (PanIN) (2/10, 20%); low-grade intraductal papillary mucinous neoplasm (IPMN) (2/10, 20%); chronic pancreatitis (4/10, 40%); and unknown (1/10, 10%). PDAC, PanIN and IPMN were finally diagnosed by surgical specimens. Malignancy was observed in three cases (30%), where one (PDAC) was classified as stage I, and two (PanIN) as stage 0, according to the 8th edition of the Union for International Cancer Control (UICC) clinical staging system. During ERCP, carbohydrate antigen 19-9 (CA19-9) level ranged from 2.9 to 37.4 U/mL (median, 5.5 U/mL), and carcinoembryonic antigen (CEA) level ranged from 1.8 to 3.5 ng/mL (median, 2.9 ng/mL) (Table 1). In the bile group, the median age of the patients was 69.5 years (range, 35–85 years), and 28 (70%) were men. Pancreaticobiliary diseases include perihilar extrahepatic cholangiocarcinoma (perihilar EHCC) (16/40, 40%); distal EHCC (8/40, 20%); PDAC (5/40, 13%); intrahepatic cholangiocarcinoma (IHCC) (2/40, 5%); gallbladder carcinoma (GBC) (1/40, 2.5%); and benign bile duct disease (as control) (8/40, 20%). Eight perihilar EHCC, four distal EHCC and one PDAC were finally diagnosed by surgical specimens. Thirty-two cases were malignant, of which 5, 14, 6 and 7 cases were classified as stages I, II, III and IV, respectively, according to the 8th edition of the UICC clinical staging system. During ERCP, CA19-9 level ranged from 2.4 to 70,632 U/mL (median, 53.8 U/mL), and CEA level ranged from 1.8 to 541.2 ng/mL (median, 3.1 ng/mL) (Table 2).

### 3.2. Pancreatic Juice and Bile cfDNA Fragment Size Distributions and cfDNA Concentrations

**Figure 1:**
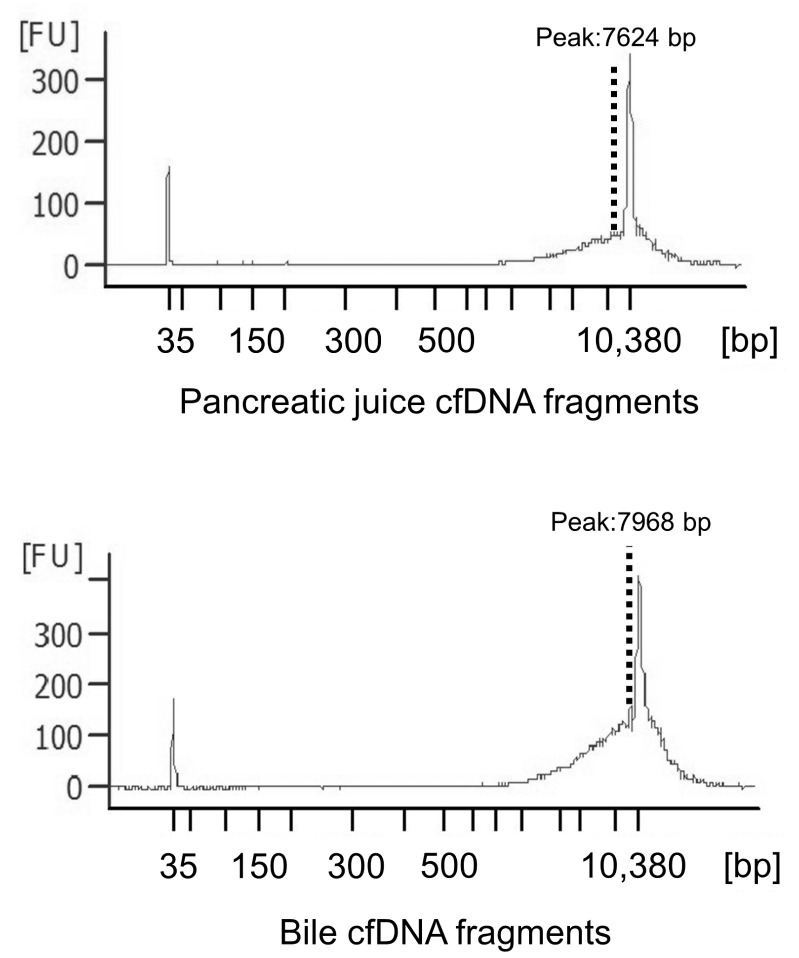
Cell-free DNA (cfDNA) fragment distributions in pancreatic juice and bile. The electoropherograms revealed that all the samples had similar fragment lengths of approximately 8000–10,000 bp.

**Figure 2:**
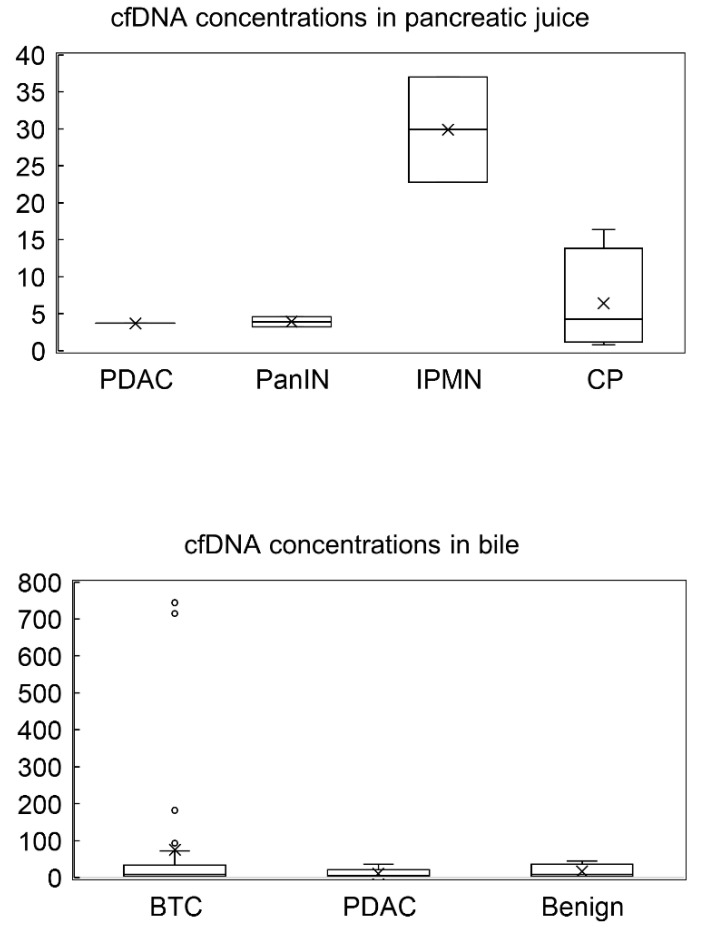
cfDNA concentrations in pancreatic juice and bile. cfDNA concentrations in pancreatic juice was higher in the IPMN group than in other groups (PDAC, PanIN and CP groups), while cfDNA concentrations in bile were similar in all the groups. PDAC, pancreatic ductal adenocarcinoma; PanIN, pancreatic intraepithelial lesion; IPMN, intraductal papillary mucinous neoplasm; CP, chronic pancreatitis; BTC, biliary tract cancer; Benign, benign stricture. x means the median and each circle means each value.

The electropherograms revealed that all samples had similar distributions and had approximately 8000–10,000 bp fragments (Figure 1), although plasma cfDNA has been reported to be distributed at approximately 150–200 bp [29]. This result indicates that long cfDNA fragments are more prevalent in the pancreatic juice and bile than in the plasma. The cfDNA concentrations in pancreatic juice ranged from 0.8 to 37 ng/μL and were higher in the IPMN group than in the PDAC, PanIN and CP groups (not statistically evaluated because of small sample size). Meanwhile, cfDNA concentrations in bile ranged from 1.4 to 715 ng/μL and were higher than that in pancreatic juice and similar in all the groups (*p* = 0.419) (Table 3 and Table 4, Figure 2).

### 3.3. cfDNA Mutational and Cytological Analyses in the Pancreatic Juice Group

**Figure 3:**
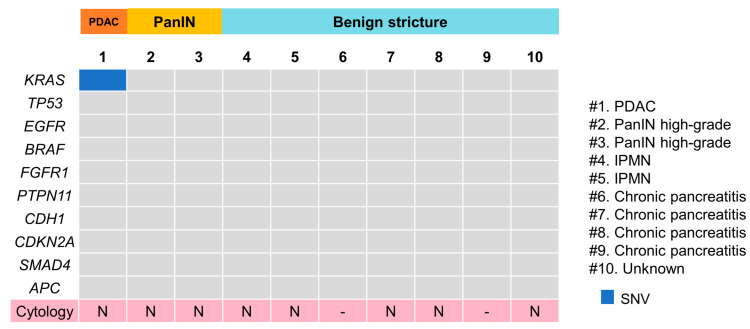
Pathogenic gene alteration profile in pancreatic juice cfDNA. PDAC, pancreatic ductal adenocarcinoma; PanIN, pancreatic intraepithelial lesion; IPMN, intraductal papillary mucinous neoplasm. *KRAS* mutation alone was detected in one PDAC case. (#) means case number.

The Cancer Hotspot Panel v2 analysis showed an average total read of 602,158. The average sequencing depth of coverage was ×2322. Among the 10 patients, only one single nucleotide variant (SNV) was identified in one PDAC patient (10%), in which genomic profiles revealed pathogenic alterations in *KRAS* (Figure 3). Pancreatic juice cytology performed in eight patients, including one PDAC case, was negative. The sensitivity, specificity and positive and negative predictive values of cfDNA analyses of pancreatic juice were 33%, 100%, 100% and 71.4%, respectively; those of the cytological analyses were 0%, 100%, 0% and 62.5%, respectively. The sensitivity and specificity were not significantly different between cfDNA analyses and the cytological analyses.

### 3.4. cfDNA Mutational and Cytological Analyses in the Bile Group

**Figure 4:**
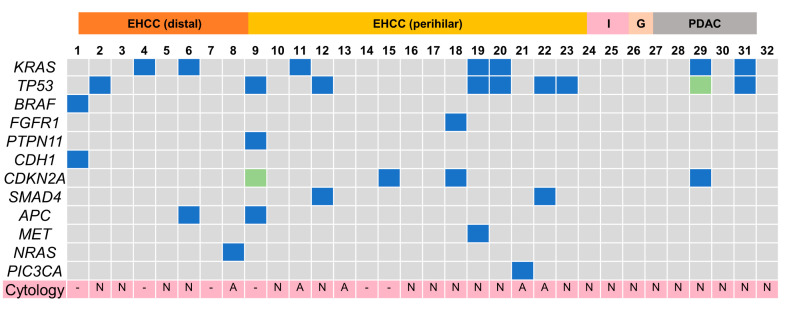
Pathogenic gene alteration profile in bile cfDNA of the patients with pancreaticobiliary malignancy. Multiple genetic mutations were detected except for intrahepatic cholangiocarcinoma and gallbladder cancer. EHCC, extrahepatic cholangiocarcinoma; I, intrahepatic cholangiocarcinoma; G, gallbladder cancer; PDAC, pancreatic ductal adenocarcinoma; N, negative; A, adenocarcinoma; -, not examined; SNV, single nucleotide variance.

**Figure 5:**
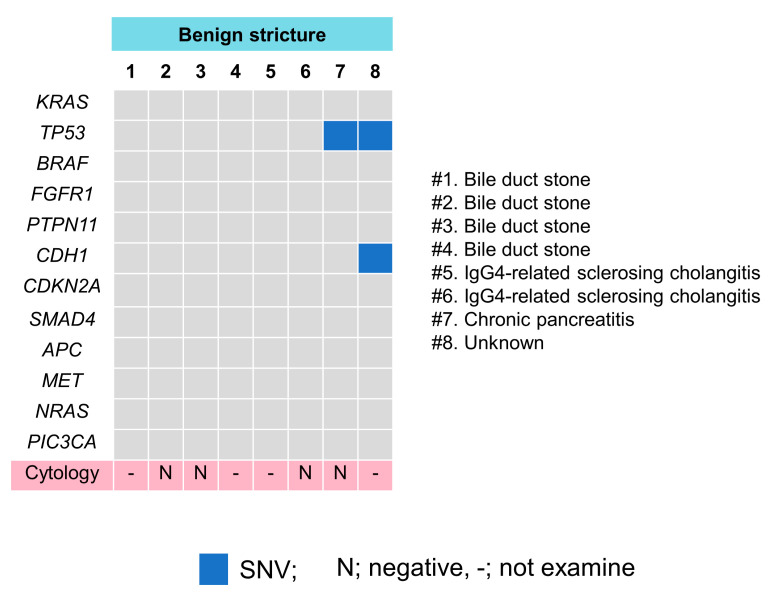
Pathogenic gene alteration profile in bile cfDNA of the patients with benign biliary stricture. *TP53* and *CDH1* mutations were detected in two patients. (#) means case number.

The Cancer Hotspot Panel v2 analysis showed an average total read of 550,497. The average sequencing depth of coverage was ×2235. In the bile group, 32 SNVs and 2 insertions and deletions (INDELs) were identified in 12 genes (Figure 4 and Figure 5, and Table 5). Pathogenic alterations were identified in 19 (47.5%) of the 40 patients and 17 (53.1%) of the 32 patients with malignancies. Genomic profiling revealed pathogenic alterations in *TP53* (9/32, 28%); *KRAS* (7/32, 22%); *CDKN2A* (4/32, 12.5%); *SMAD4* (2/32, 6.3%); *APC* (2/32, 6.3%); *BRAF* (1/32, 3.1%); *FGFR1* (1/32, 3.2%); *PTPN11* (1/32, 3.2%); *CDH1* (1/32, 3.2%); *MET* (1/32, 3.2%); *NRAS* (1/32, 3.2%); and *PIC3CA* (1/32, 3.2%). However, alterations in *TP53* (2/8, 25%) and *CDH1* (1/8, 12.5%) were positive in two patients with benign diseases during examinations; these were an unknown case and a chronic pancreatitis (CP) case, respectively, neither of which was malignant within the observational period. Bile cytology was performed on 30 patients, and the sensitivity, specificity and positive and negative predictive values of the cytological analyses were 19%, 100%, 100% and 16%, respectively, while those of the bile cfDNA analyses were 53%, 75%, 89.5% and 28.6%, respectively. The sensitivity of the bile cfDNA analysis was significantly higher than that of the cytological analysis (*p* = 0.016), whereas the specificities of them were similar (*p* = 0.48).

### 3.5. Pathogenic Gene Alterations in Each Cancer Site in the Bile Group

**Figure 6:**
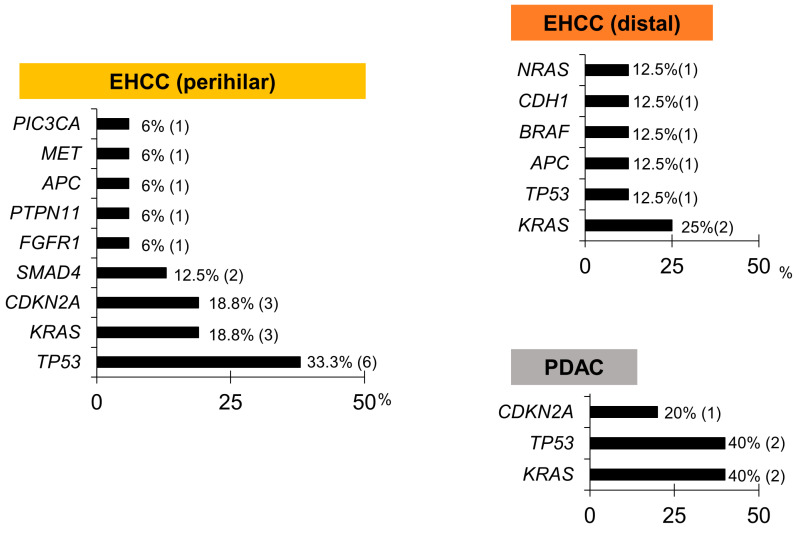
Pathogenic gene alteration frequencies in bile cfDNA, according to the cancer site. *KRAS* and *TP53* mutations were the most frequent, followed by *CDKN2A*.

In perihilar EHCC, pathogenic gene alterations were identified in 10 patients (62.5%), and alterations in *TP53* (37.5%) were frequently identified as pathogenic. In distal EHCC and PDAC, pathogenic gene alterations were identified in five (62.5%) and two patients (40%), respectively. In IHCC and GBC, pathogenic gene alterations were not identified (Figure 6).

## 4. Discussion

Here, we successfully isolated long cfDNA from pancreatic juice and bile, which had a different length than that obtained from the plasma previously reported and demonstrated that cfDNA could be extracted from pancreatic juice and bile of patients with pancreatic/bile duct stricture for genetic analysis.

Previous studies have reported that plasma cfDNA contains a high percentage of short fragments of 150–200 bp. However, in this study, pancreatic juice and bile cfDNA fragments were very long, similar to those indicated in previous reports [23,26]. The differences in length between plasma cfDNA and pancreatic juice/bile cfDNA could be attributed to the differences in the type and activity of DNA restriction endonucleases in both [23]. Long cfDNA fragments can be beneficial for high-sensitivity detection of gene alterations, and this concept can be applied to newer DNA sequencing technology with long-read sequencing [30]. Meanwhile, the cfDNA concentration in bile was higher than that in pancreatic juice in the present study. In this study cohort, more patients in the bile group had more advanced tumors than in the pancreatic juice group, which may have caused the difference in the cfDNA concentrations between the two groups. Furthermore, cfDNA concentrations in pancreatic juice with IPMN were higher than those in pancreatic juice with other pancreatic diseases. IPMN is characterized by production of much mucus, which also contains cfDNA and exosome with DNA [31] derived from neoplastic cells. There has been no report comparing the amount of cfDNA in pancreatic juice among PDAC, IPMN and others, and only two IPMN cases were enrolled in the present study; however, the amount of cfDNA might be proportional to the amount of mucus. Meanwhile, according to the report by Mateos et al. [20], the ratio of tumor-derived cfDNA in pancreatic juice did not correlate with IPMN grade.

This study also revealed that the sensitivities of cfDNA analyses of pancreatic juice and bile for malignancies were higher than those of cytological assessments (pancreatic juice, 33% vs. 0%; bile, 53% vs. 19%). These results suggest that cfDNA analysis of pancreatic juice and bile with cell-derived materials is an available and complementary diagnostic tool, in addition to their cytology.

In the pancreatic juice group, *KRAS* mutations were detected in stage I PDAC; however, no mutations were detected in high-grade PanIN cases. Genetic alterations in *KRAS*, *CDKN2A*, *TP53* and *SMAD4* were reported to be involved stepwise in PanIN progress [32,33,34,35]. Theoretically, cfDNA containing these genetic alterations would be present in the pancreatic juice; however, this was not detected in this study, probably due to the lower threshold of mutant allele frequency (MAF) of 2%, which was higher than that of previous bile cfDNA reports [22,26]. Few reports are available on multiple gene alterations of cfDNA in pancreatic juice using NGS. Mateos et al. [20] searched for all exome alterations with the lower threshold of MAF of 5%, including copy number variation and representative *KRAS*, *GNAS*, *RNF43* and *TP53* SNVs and INDELs with pancreatic juice cfDNA in various IPMN. They revealed that almost all samples harbored some somatic mutation, while *KRAS* and *GNAS* mutations were detected in 44% and 31% of the samples, respectively, consistent with our analyses, which are time- and cost-efficient. Ginesta et al. [36] assessed the methylation status of *EN-1*, *HRH2*, *SPARC*, *APC* and *CDH13* gene promoters with pancreatic juice cfDNA and indicated that *APC* methylation was detected in ampullary carcinoma (76%) and IPMN (80%) but was occasionally observed in CP (7%). Meanwhile, positive predictive values for malignancy were low, and the differential diagnosis was difficult (a drawback of their study).

In the bile group, no mutations were detected in IHCC and GBC. Previous studies have detected bile cfDNA mutations with high sensitivity in patients diagnosed with IHCC and GBC using a more comprehensive gene panel with 150–160 genes and a high-spec sequencer with 0.15 to 0.1% of the lower limit of MAF [22,26]. Among benign diseases, genetic alterations were detected in two cases of CP and unknown origin. Although these might be false positives, a previous study reported that four cases with an initial diagnosis of benign disease with positive gene alterations developed PDAC during the follow-up period [26]. Therefore, patients with an initial diagnosis of benign disease and positive gene alterations should be continuously and longitudinally surveyed with caution.

This study had some limitations. First, the lower detection limit of MAF of the DNA sequencer was 2%, which might be insufficient for detecting cfDNA mutations in bile and pancreatic juice compared with previous reports [22,26]. Second, only a few patients with pancreatic (*n* = 10) and benign biliary diseases (*n* = 40) were enrolled in this study, which means a small sample size and disease bias; this may prevent adequate validation of mutation measurements in the pancreatic juice and bile. Third, the oncogene panel with 50 genes used in this study did not cover all relevant mutational genes in the pancreaticobiliary field, such as *ATM*, *ARID1A/2A*, *BRCA* and *RNF43*. Fourth, for simplicity, we defined malignancy as the one or more gene mutations with the 50-gene panel. Considering a multistep carcinogenesis theory, multiple gene mutations can accumulate before cancerization, and a single gene mutation may be detected in noncancerous or precancerous cells. Thus, the definition of malignancy with cfDNA analysis requires reexamination with an increased number of cases and a comprehensive gene panel with more genes.

In clinical practice, cfDNA analysis would enable a complementary diagnosis of malignancy/benignancy in cases with indeterminate cytology of pancreatic juice and bile or help for prediction of future malignancy based on genetic mutation. We believe that further cases will confirm the validity of the cfDNA analysis in the future.

Overall, pancreatic juice and bile cfDNA as a novel liquid biopsy tool is feasible and available for detecting multiple gene mutations using NGS and promotes the diagnosis of pancreaticobiliary strictures.

## Figures and Tables

**Table 1:** Characteristics of 10 patients in the pancreatic juice group.

Characteristics	*N*
Age, median (range), y	65.5 (47–80)
Sex, *n* (%)	
Male	7 (70)
Female	3 (30)
Details of pancreatic disease, *n* (%)	
Malignant disease	
PDAC	1 (10)
PanIN (High grade)	2 (20)
Benign disease as control	
IPMN	2 (20)
Chronic pancreatitis	4 (40)
Unknown	1 (10)
UICC stage in malignant (*n* = 3), *n* (%)	
0 (PanIN)	2 (67)
I (PDAC)	1 (33)
Tumor marker level at the time of ERCP	
CA19-9, median (range) U/mL	5.5 (2.9–37.4)
CEA, median (range) ng/mL	2.9 (1.8–3.5)

Abbreviations: PDAC, pancreatic ductal adenocarcinoma; PanIN, pancreatic intraepithelial neoplasm; IPMN, intraductal papillary mucinous neoplasm; ERCP, endoscopic retrograde cholangiopancreatography.

**Table 2:** Characteristics of 40 patients in the bile group.

Characteristics	*N*
Age, median (range), y	69.5 (35–85)
Sex, *n* (%)	
Male	28 (70)
Female	12 (30)
Details of bile disease, *n* (%)	
Malignant disease	
Perihilar EHCC	16 (40)
Distal EHCC	8 (20)
PDAC	5 (13)
IHCC	2 (5)
GBC	1 (2.5)
Benign disease as control	
Bile duct stone	4 (10)
IgG4-related sclerosing cholangitis	2 (5)
Chronic pancreatitis	1 (2.5)
Unknown	1 (2.5)
UICC stage in malignant (total 32), *n* (%)	
I	5 (15.6)
II	14 (43.7)
III	6 (18.8)
IV	7 (21.9)
Jaundice (total Bilirubin > 2mg/d), *n* (%)	
+	19 (48)
−	21 (52)
Tumor marker level at the time of ERCP	
CA19-9, median (range) U/mL	53.8 (2.4–70,632)
CEA, median (range) ng/mL	3.1 (1.8–541.2)

Abbreviations: EHCC, extrahepatic cholangiocarcinoma; PDAC, pancreatic ductal adenocarcinoma; IHCC, intrahepatic cholangiocarcinoma; GBC: gallbladder carcinoma; ERCP, endoscopic retrograde cholangiopancreatography. (+) means presence of jaundice; (−) means absence of jaundice.

**Table 3:** Concentrations of cfDNA, genomic DNA (gDNA) and library in the pancreatic juice group.

No.	Disease	cfDNA Concentration (ng/μL)	gDNA Concentration (ng/μL)	Library Concentration cfDNA (pM)	Library Concentration gDNA (pM)	UICC Stage
1	PDAC	3.7	28.59	1995.8	360.6	I
2	PanIN	4.6	31.264	1681.5	1538.7	0
3	PanIN	3.26	16.494	494.9	1413.5	0
4	IPMN	22.8	53.829	952.6	2969.2	
5	IPMN	37	17.299	856.6	1163.9	
6	CP	2.3	46.752	1002.2	569.7	
7	CP	6.28	19.365	1148.2	1213.4	
8	CP	0.814	17.122	643.1	4742	
9	CP	16.4	35.254	913.9	806.5	
10	Unknown	10.9	34.723	1821.7	11,473.3	

Abbreviations: PDAC, pancreatic ductal adenocarcinoma; PanIN, pancreatic intraepithelial neoplasm; IPMN, intraductal papillary mucinous neoplasm; CP, chronic pancreatitis.

**Table 4:** Concentrations of cfDNA, genomic DNA (gDNA) and library in the bile group.

No	Disease	Jaundice	cfDNA Concentration (ng/μL)	gDNA Concentration (ng/μL)	Library Concentration cfDNA (pM)	Library Concentration gDNA (pM)	UICC Stage
1	dEHCC	−	2.44	11.896	392.7	3884.2	II
2	dEHCC	+	6.52	53.224	2802.5	967.6	II
3	dEHCC	−	72.8	27.75	444.9	5141	II
4	dEHCC	+	13.4	21.273	1111.6	1365.8	II
5	dEHCC	−	182.4	40.905	389.7	360.6	I
6	dEHCC	−	19.7	24.143	1668	365.1	I
7	dEHCC	+	9	17.537	2822.5	1333.4	IV
8	dEHCC	+	6.36	30.25	2019.4	1104.1	I
9	pEHCC	−	20	37.624	635.9	928.4	III
10	pEHCC	−	8.02	39.199	1115.1	639	II
11	pEHCC	+	13.9	18.688	2752.8	1576	III
12	pEHCC	+	5.06	42.843	712.8	584	IV
13	pEHCC	−	4.42	24.224	843.9	354.2	II
14	pEHCC	+	715.2	80.663	1387.1	2874.2	II
15	pEHCC	−	4.26	29.95	1729.2	912.3	II
16	pEHCC	+	1.74	38.77	2260.8	851	IV
17	pEHCC	−	35	37.49	882.3	775.4	II
18	pEHCC	−	744	44.423	717.6	1048.8	IV
19	pEHCC	+	2.92	48.875	1066.4	1455.4	III
20	pEHCC	+	6.08	33.725	1190.3	840.8	III
21	pEHCC	+	13.5	36.009	536.2	860.6	IV
22	pEHCC	−	25	23.852	1204.6	4742	II
23	pEHCC	−	3.52	22.667	2780.8	604.8	II
24	pEHCC	+	4.22	46.813	1579.2	2050.1	IV
25	IHCC	−	33.8	32.329	377.4	847.8	III
26	IHCC	+	5.5	24.225	759.5	603.4	II
27	GBC	−	93.4	32.078	742.9	1358.3	IV
28	PDAC	+	4.88	38.456	977.8	2215.8	II
29	PDAC	+	4.18	29.496	1616.1	1343.1	II
30	PDAC	+	7.64	24.75	1128.4	887.3	II
31	PDAC	+	1.4	22.868	2161.1	185.2	II
32	PDAC	−	36.4	30.669	2002.7	905.2	III
33	Stone	+	14.3	25.403	940.6	412.1	
34	Stone	−	6.34	28.962	2190.8	2267.7	
35	Stone	−	3.88	20.325	1009.5	339.4	
36	Stone	−	1.36	29.85	2134.7	345	
37	IgG4-SC	+	11.4	26.902	293.6	1811.9	
38	IgG4-SC	−	45	50.492	863.6	1474.7	
39	CP	−	44.4	46.752	923.4	569.7	
40	Unknown	−	7.72	30.351	4930.9	2875.6	

Abbreviations: dEHCC, distal extrahepatic cholangiocarcinoma; pEHCC, perihilar extrahepatic cholangiocarcinoma; IHCC, intrahepatic cholangiocarcinoma; GBC, gallbladder carcinoma; PDAC, pancreatic ductal adenocarcinoma; Stone, bile duct stone; CP, chronic pancreatitis; IgG4-SC, IgG4-related sclerosing cholangitis. (+) means presence of jaundice; (−) means absence of jaundice.

**Table 5:** Pathogenic alterations in the bile group.

No	Disease	Cytology	Pathogenic Alteration	Variant Type	Locus
1	dEHCC	-	*BRAF*	SNV	chr7:140453149
			*CDH1*	SNV	chr16:68847276”
2	dEHCC	N	*TP53*	SNV	chr17:7574017
3	dEHCC	N	None	None	None
4	dEHCC	-	*KRAS*	SNV	chr12:25398280
5	dEHCC	N	None	None	None
6	dEHCC	N	*KRAS*	SNV	chr12:25398280
			*APC*	SNV	chr5:112175951
7	dEHCC	-	None	None	None
8	dEHCC	A	*NRAS*	SNV	chr1:115256528
9	pEHCC	-	*TP53*	SNV	chr17:7578256
			*PTPN11*	SNV	chr12:112888210
			*CDKN2A*	INDEL	chr9:21971168
			*APC*	SNV	chr5:112175639
10	pEHCC	N	None	None	None
11	pEHCC	A	*KRAS*	SNV	chr12:25398280
12	pEHCC	N	*TP53*	SNV	chr17:7578526
			*SMAD4*	SNV	chr18:48591821
13	pEHCC	A	None	None	None
14	pEHCC	-	None	None	None
15	pEHCC	-	*CDKN2A*	SNV	chr9:21971209
16	pEHCC	N	None	None	None
17	pEHCC	N	None	None	None
18	pEHCC	N	*FGFR1*	SNV	chr8:38282147
			*CDKN2A*	SNV	chr9:21971036
19	pEHCC	N	*KRAS*	SNV	chr12:25380275
			*TP53*	SNV	chr17:7578368
			*MET*	SNV	chr7:116340262
20	pEHCC	N	*KRAS*	SNV	chr12:25380275
			*TP53*	SNV	chr17:7578368
21	pEHCC	A	*PIC3CA*	SNV	chr3:178952074
22	pEHCC	A	*TP53*	SNV	chr17:7577557
			*SMAD4*	SNV	chr18:48591931
23	pEHCC	N	*TP53*	SNV	chr17:7577528
24	pEHCC	N	None	None	None
25	IHCC	N	None	None	None
26	IHCC	N	None	None	None
27	GBC	N	None	None	None
28	PDAC	N	None	None	None
29	PDAC	N	*KRAS*	SNV	chr12:25398280
			*TP53*	INDEL	chr17:7577553
			*CDKN2A*	SNV	chr9:21971101
30	PDAC	N	None	None	None
31	PDAC	N	*KRAS*	SNV	chr12:25398280
			*TP53*	SNV	chr17:7574002
32	PDAC	N	None	None	None
33	Stone	-	None	None	None
34	Stone	N	None	None	None
35	Stone	N	None	None	None
36	Stone	-	None	None	None
37	IgG4-SC	-	None	None	None
38	IgG4-SC	N	None	None	None
39	CP	N	*TP53*	SNV	chr17:7577100
40	Unknown	-	*TP53*	SNV	chr17:7578475
			*CDH1*	SNV	chr16:68847240

Abbreviations: dEHCC, distal extrahepatic cholangiocarcinoma; pEHCC, perihilar extrahepatic cholangiocarcinoma; IHCC, intrahepatic cholangiocarcinoma; GBC, gallbladder carcinoma; PDAC, pancreatic ductal adenocarcinoma; Stone, bile duct stone; CP, chronic pancreatitis; IgG4-SC, IgG4-related sclerosing cholangitis. (-) means not examine; N, negative; A, adenocarcinoma.

## Data Availability

The data presented in this study are available on request from the corresponding author. The data are not publicly available due to privacy laws.

## References

[1] Mizrahi J.D., Surana R., Valle J.W., Shroff R.T. (2020). Pancreatic cancer. Lancet.

[2] Valle J.W., Kelley R.K., Nervi B., Oh D.Y., Zhu A.X. (2021). Biliary tract cancer. Lancet.

[3] Groot V.P., van Santvoort H.C., Rombouts S.J., Hagendoorn J., Borel Rinkes I.H., van Vulpen M., Herman J.M., Wolfgang C.L., Besselink M.G., Molenaar I.Q. (2017). Systematic review on the treatment of isolated local recurrence of pancreatic cancer after surgery; re-resection, chemoradiotherapy and SBRT. HPB.

[4] Endo I., Gonen M., Yopp A.C., Dalal K.M., Zhou Q., Klimstra D., D’Angelica M., DeMatteo R.P., Fong Y., Schwartz L. (2008). Intrahepatic cholangiocarcinoma: Rising frequency, improved survival, and determinants of outcome after resection. Ann. Surg..

[5] Kleeff J., Korc M., Apte M., La Vecchia C., Johnson C.D., Biankin A.V., Neale R.E., Tempero M., Tuveson D.A., Hruban R.H. (2016). Pancreatic cancer. Nat. Rev. Dis Primers.

[6] Khan S.A., Emadossadaty S., Ladep N.G., Thomas H.C., Elliott P., Taylor-Robinson S.D., Toledano M.B. (2012). Rising trends in cholangiocarcinoma: Is the ICD classification system misleading us?. J. Hepatol..

[7] Eloubeidi M.A., Chen V.K., Eltoum I.A., Jhala D., Chhieng D.C., Jhala N., Vickers S.M., Wilcox C.M. (2003). Endoscopic ultrasound-guided fine needle aspiration biopsy of patients with suspected pancreatic cancer: Diagnostic accuracy and acute and 30-day complications. Am. J. Gastroenterol..

[8] Burnett A.S., Calvert T.J., Chokshi R.J. (2013). Sensitivity of endoscopic retrograde cholangiopancreatography standard cytology: 10–y review of the literature. J. Surg. Res..

[9] Navaneethan U., Njei B., Lourdusamy V., Konjeti R., Vargo J.J., Parsi M.A. (2015). Comparative effectiveness of biliary brush cytology and intraductal biopsy for detection of malignant biliary strictures: A systematic review and meta–analysis. Gastrointest. Endosc..

[10] Hatfield A.R., Smithies A., Wilkins R., Levi A.J. (1976). Assessment of endoscopic retrograde cholangio-pancreatography (ERCP) and pure pancreatic juice cytology in patients with pancreatic disease. Gut.

[11] Kameya S., Kuno N., Kasugai T. (1981). The diagnosis of pancreatic cancer by pancreatic juice cytology. Acta Cytol..

[12] Goodale R.L., Gajl-Peczalska K., Dressel T., Samuelson J. (1981). Cytologic studies for the diagnosis of pancreatic cancer. Cancer.

[13] Nakaizumi A., Tatsuta M., Uehara H., Yamamoto R., Takenaka A., Kishigami Y., Takemura K., Kitamura T., Okuda S. (1992). Cytologic examination of pure pancreatic juice in the diagnosis of pancreatic carcinoma. The endoscopic retrograde intraductal catheter aspiration cytologic technique. Cancer.

[14] Gormally E., Caboux E., Vineis P., Hainaut P. (2007). Circulating free DNA in plasma or serum as biomarker of carcinogenesis: Practical aspects and biological significance. Mutat. Res..

[15] Ignatiadis M., Sledge G.W., Jeffrey S.S. (2021). Liquid biopsy enters the clinic—implementation issues and future challenges. Nat. Rev. Clin. Oncol..

[16] Molina-Vila M.A., de-Las-Casas C.M., Bertran-Alamillo J., Jordana-Ariza N., González-Cao M., Rosell R. (2015). cfDNA analysis from blood in melanoma. Ann. Transl. Med..

[17] Hadano N., Murakami Y., Uemura K., Hashimoto Y., Kondo N., Nakagawa N., Sueda T., Hiyama E. (2016). Prognostic value of circulating tumour DNA in patients undergoing curative resection for pancreatic cancer. Br. J. Cancer.

[18] Waddell N., Pajic M., Patch A.M., Chang D.K., Kassahn K.S., Bailey P., Johns A.L., Miller D., Nones K., Quek K. (2015). Whole genomes redefine the mutational landscape of pancreatic cancer. Nature.

[19] Nakamura H., Arai Y., Totoki Y., Shirota T., Elzawahry A., Kato M., Hama N., Hosoda F., Urushidate T., Ohashi S. (2015). Genetic spectra of billiary tract cancer. Nat. Genet..

[20] Mateos R.N., Nakagawa H., Hirono S., Takano S., Fukasawa M., Yanagisawa A., Yasukawa S., Maejima K., Oku-Sasaki A., Nakano K. (2019). Genomic analysis of pancreatic juice DNA assesses malignant risk of intraductal papillary mucinous neoplasm of pancreas. Cancer Med..

[21] Okada T., Iwano H., Ono Y., Karasaki H., Sato T., Yamada M., Omori Y., Sato H., Hayashi A., Kawabata H. (2018). Utility of ‘liquid biopsy’ using pancreatic juice for early detection of pancreatic cancer. Endosc. Int. Open.

[22] Kinugasa H., Nouso K., Ako S., Dohi C., Matsushita H., Matsumoto K., Kato H., Okada H. (2018). Liquid biopsy of bile for the molecular diagnosis of gallbladder cancer. Cancer Biol. Ther..

[23] Shen N., Zhang D., Yin L., Qiu Y., Liu J., Yu W., Fu X., Zhu B., Xu X., Duan A. (2019). Bile cell-free DNA as a novel and powerful liquid biopsy for detecting somatic variants in biliary tract cancer. Oncol. Rep..

[24] Driescher C., Fuchs K., Haeberle L., Goering W., Frohn L., Opitz F.V., Haeussinger D., Knoefel W.T., Keitel V., Esposito I. (2020). Bile-based cell-free DNA analysis is a reliable diagnostic tool in pancreatobiliary cancer. Cancers.

[25] Han J.Y., Ahn K.S., Kim T.S., Kim Y.H., Cho K.B., Shin D.W., Baek W.K., Suh S.I., Jang B.C., Kang K.J. (2021). Liquid biopsy from bile-circulating tumor DNA in patients with biliary tract cancer. Cancers.

[26] Arechederra M., Rullán M., Amat I., Oyon D., Zabalza L., Elizalde M., Latasa M.U., Mercado M.R., Ruiz-Clavijo D., Saldaña C. (2022). Next-generation sequencing of bile cell-free DNA for the early detection of patients with malignant biliary strictures. Gut.

[27] Saunders C.T., Wong W.S., Swamy S., Becq J., Murray L.J., Cheetham R.K. (2012). Strelka: Accurate somatic small variant calling from sequenced tumor-normal sample pairs. Bioinformatics.

[28] Kanda Y. (2013). Investigation of the freely available easy-to-use software ‘EZR’ for medical statistics. Bone Marrow Transplant..

[29] Yang N., Li Y., Liu Z., Qin H., Du D., Cao X., Cao X., Li J., Li D., Jiang B. (2018). The characteristics of ctDNA reveal the high complexity in matching the corresponding tumor tissues. BMC Cancer.

[30] Sakamoto Y., Sereewattanawoot S., Suzuki A. (2020). A new era of long-read sequencing for cancer genomics. J. Hum. Genet..

[31] Yang K.S., Ciprani D., O’Shea A., Liss A.S., Yang R., Fletcher-Mercaldo S., Mino-Kenudson M., Castillo C.F.D., Weissleder R. (2021). Extracellular vesicle analysis allows for identification of invasive IPMN. Gastroenterology.

[32] Iacobuzio-Donahue C.A., Velculescu V.E., Wolfgang C.L., Hruban R.H. (2012). Genetic basis of pancreas cancer development and progression: Insights from whole-exome and whole-genome sequencing. Clin. Cancer Res..

[33] Boschman C.R., Stryker S., Reddy J.K., Rao M.S. (1994). Expression of p53 protein in precursor lesions and adenocarcinoma of human pancreas. Am. J. Pathol..

[34] Hruban R.H., Wilentz R.E., Kern S.E. (2000). Genetic progression in the pancreatic ducts. Am. J. Pathol..

[35] Hezel A.F., Kimmelman A.C., Stanger B.Z., Bardeesy N., Depinho R.A. (2006). Genetics and biology of pancreatic ductal adenocarcinoma. Genes Dev..

[36] Ginesta M.M., Diaz-Riascos Z.V., Busquets J., Pelaez N., Serrano T., Peinado M.À., Jorba R., García-Borobia F.J., Capella G., Fabregat J. (2016). APC promoter is frequently methylated in pancreatic juice of patients with pancreatic carcinomas or periampullary tumors. Oncol. Lett..

